# Youth perception of difficulty accessing cannabis following cannabis legalization and during the early and ongoing stages of the COVID-19 pandemic: repeat cross-sectional and longitudinal data from the COMPASS study

**DOI:** 10.1186/s13690-023-01224-x

**Published:** 2023-12-15

**Authors:** Scott T. Leatherdale, Angelica Amores, Richard E. Bélanger, Kate Battista, Karen A. Patte, Ying Jiang

**Affiliations:** 1 School of Public Health Sciences, University of Waterloo, 200 University Avenue, Waterloo, ON N2L 3G1 Canada; 2 VITAM - Centre de Recherche en Santé Durable, Université Laval, Quebec City, Canada; 3 Department of Pediatrics, Faculty of Medicine, Université Laval, Quebec City, Canada; 4 Department of Health Sciences, Faculty of Applied Health Sciences, Brock University, St. Catherines, Canada; 5 Applied Research Division, Public Health Agency of Canada, Ottawa, Canada

**Keywords:** COVID-19, Youth, Cannabis use, Cannabis access, Longitudinal

## Abstract

**Background:**

Very little research has examined how perceptions of cannabis access among underage youth in Canada have changed since cannabis was legalized and since the onset of the COVID-19 pandemic. As such, this paper examines the effect of the early and ongoing stages of the COVID-19 pandemic period on youth perceptions of cannabis access over time since the onset of the Cannabis Act in 2018 in a large sample of Canadian youth.

**Methods:**

Using data from the COMPASS study (T1:2018/19, T2:2019/20, T3:2020/21), we used both repeat cross-sectional data [T1 (*n* = 38,890), T2 (*n* = 24,109), and T3 (*n* = 22,795)] to examine overall trends in perceptions of cannabis access, and sequential cohort longitudinal data [*n* = 4,677 students linked from T1 to T3] to examine the differential changes in perceptions of cannabis access among students over time.

**Results:**

In the cross-sectional sample, the frequency of students reporting that cannabis was easy to access decreased by 26.7% from T1 (51.0%) to T3 (37.4%), although respondents who have used cannabis were more likely to report access was easy. In the longitudinal sample, perceptions of cannabis access being easy increased over time, especially among cannabis users. Perceived ease of access appears to have been slightly impeded during the initial pandemic period but rebounded during the ongoing pandemic period.

**Conclusions:**

While the prevalence of youth reporting that cannabis is easy to access has declined since legalization and throughout the early and ongoing pandemic periods, a substantial number of underage youth continue to report that cannabis is easy to access. This suggest that there is an ongoing need for continued cannabis control efforts to address this issue.

**Table:** 

**Text box 1. Contributions to the literature**
• Using repeat cross-sectional and longitudinal data uniquely allows us to understand changes in prevalence and individual likelihood of perceiving cannabis as being easy to access
• The frequency of both current cannabis use and perceiving cannabis as easy to access decreased over time
• Despite legalization trying to reduce underage youth from accessing cannabis, more than a third of youth continue to report access as easy, with cannabis users being more likely to report easy access

## Background

In October 2018, the Canadian government legalized non-medical cannabis use for adults via the Cannabis Act [1]. Although there was some initial speculation and concern that this may result in an increase in cannabis use among youth, prospective evidence suggests that there was no large difference in cannabis use trends among youth pre- to post-legalization [2]. Aside from preventing criminals from profiting from cannabis and protecting public health and safety by allowing adults access to legal cannabis, the third public health goal of the Cannabis Act is to prevent underage youth from accessing cannabis [1]. Most provinces and territories set the minimum legal age to purchase cannabis products at 19, with the exception of Quebec (21 years of age) and Alberta (18 years of age) [3].

Repeat cross-sectional data from waves of the Canadian Student Tobacco, Alcohol and Drug Use Survey (CSTADS) prior to legalization suggest that the majority of underage youth (regardless of whether or not they are cannabis users) report that cannabis is easy to access (ranging between 58.5% and 63.5% from 2012 to 2016) [4]. The most common sources by which underage youth access cannabis are via family members, sharing with friends, and buying from dealers [4, 5]. Preliminary research evaluating the short-term impact of cannabis legalization suggests that Canadian youth under the legal age to purchase cannabis did not experience changes in their ability to easily access cannabis [6]. However, it is also reasonable to expect that the impact of the Cannabis Act may take some time for its effects to be realized at the population-level so ongoing examination of changes in perceptions of access over time is required.

Shortly after the World Health Organization announced that COVID-19 was a global pandemic in March 2020, researchers began to examine the impact that COVID-19 related restrictions had on youth cannabis use. The initial research from the early pandemic period suggested that cannabis use among youth populations declined slightly [7–11], although there was also evidence to suggest that cannabis use frequency increased among some established users [7, 11, 12]. To the best of our knowledge, no prospective research has examined changes in perceptions of cannabis access among youth during the early or ongoing pandemic periods. While evidence among US youth many years prior to the pandemic suggests declining perceptions of cannabis being easy to access (2002–2015), slightly more recent data from Canada suggests that youth perceptions that cannabis is easy to obtain appeared stable (2012–2016) [4, 13]. There is a need to revisit this issue in more recent timeframe surrounding the onset of the pandemic.

Considering that youth frequently report accessing cannabis from sharing with friends [5], it seems reasonable that the strict restrictions associated with the early pandemic response period (2020) should have impacted youth access to cannabis as they spent considerably less time socializing with peers. This may have changed again in the ongoing pandemic response period (2021), when many restrictions partially or completely eased hence allowing for more in-person socializing among peers. As such, the purpose of this study is to examine changes in youth perceptions of cannabis access from the post-legalization pre-pandemic period spanning to the early and then ongoing pandemic periods using an interrupted time series non-experimental design from both repeat cross-sectional and longitudinal data. A benefit of using both study designs is that the cross-sectional data allow us to explore changes in the school-level samples over time whereas the longitudinal data allow us to explore changes at the individual student-level over time, creating a more comprehensive examination of the issue.

## Methods

The COMPASS Study (COMPASS) is an ongoing prospective study [2012–2028] collecting data annually from a rolling cohort of students in grades 9 through 12 (Secondary I-V in Quebec) and the secondary schools they attend in a convenience sample of Canadian secondary schools in British Columbia, Alberta, Ontario, and Quebec [14, 15]. The student-level data are collected annually during the school year via a self-reported questionnaire across multiple content domains (including cannabis use), using an active information passive-consent protocol. Active information with passive consent protocols are essential in youth self-report research focused on substance use for producing robust results due to higher participation rates (especially among substance users) [16]. For the purpose of this study and consistent with other research using these COMPASS data spanning the pandemic period [17, 18], Time 1 (T1) data were collected in 2018/19 [paper-based survey, baseline pre-pandemic onset], Time 2 (T2) data were collected in 2019/20 [T2^a^, paper-based survey immediately before the onset of the pandemic (September 2019 to March 10, 2020); T2^b^, online survey immediately after the onset of the pandemic (April to July, 2020)], and Time 3 (T3) data were collected in 2020/21 (online survey during the 2nd and 3rd waves of the pandemic). Details on the paper-based survey and online survey and protocols are described elsewhere [19, 20], as are details on the survey questions designed to facilitate linkage of respondents within a school over time using a self-generated identification code to create our longitudinal data file [21]. All procedures were approved by the University of Waterloo Office of Research Ethics (ORE# 30118), CIUSSS de la Capitale-Nationale–Université Laval (#MP-13-2017-1264), and appropriate school board committees. A full description of the COMPASS study methods is available online [15].

### Design

**Fig. 1:**
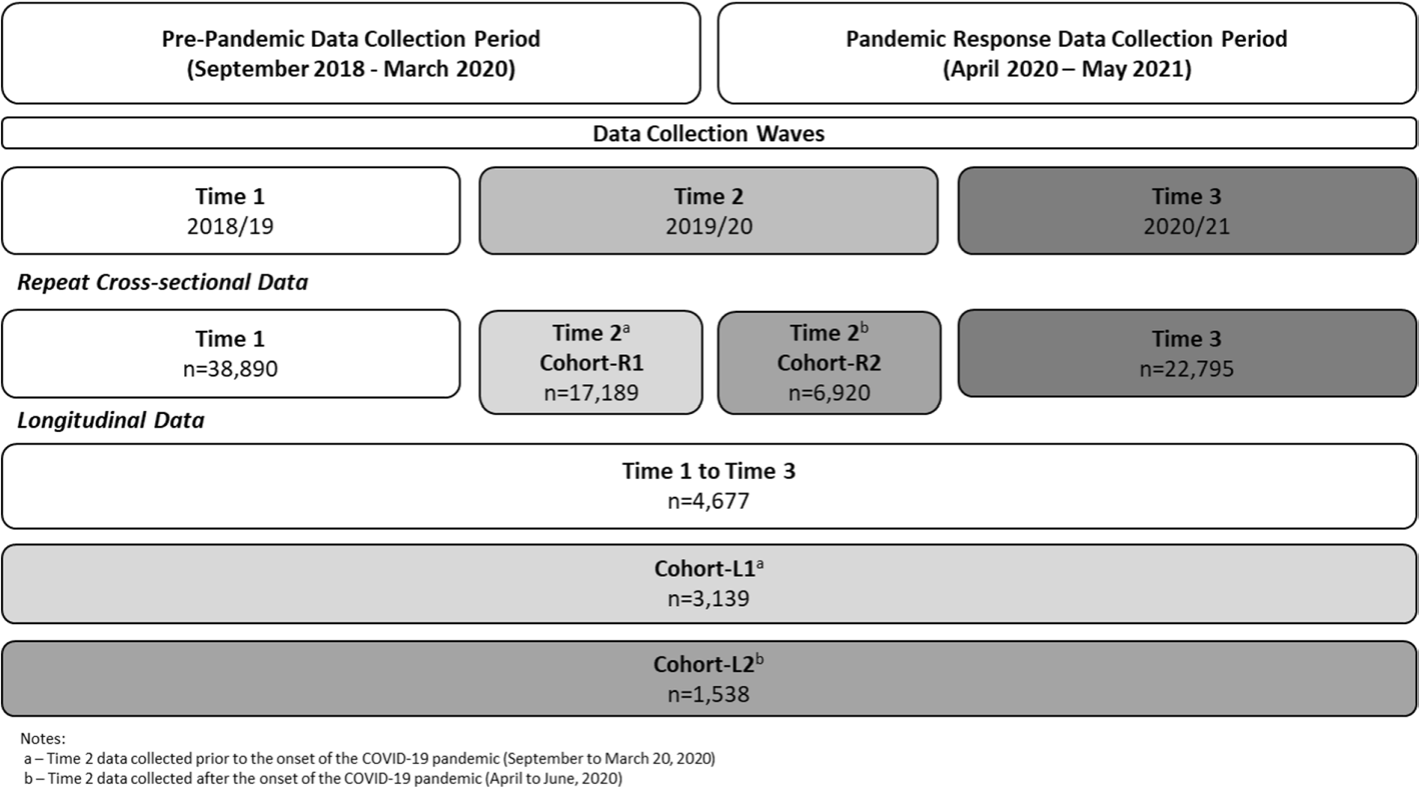
Study design and sample sizes for the repeat cross-sectional and longitudinal samples used for examining trends in youth perceptions of cannabis access prior to and during the onset and progression of the COVID-19 pandemic, COMPASS study (2018/19, 2019/20, 2020/21)

We used repeat cross-sectional data to examine overall trends in perceptions of cannabis access, and longitudinal data to examine the changes in perceptions of cannabis access among students across the onset and progression of the COVID-19 pandemic using an interrupted time series non-experimental design. This aligns with current methodological recommendations for evaluating a natural experiment such as the onset of the COVID-19 pandemic [22], and is consistent with previous research examining the impact of the pandemic on different outcomes [9, 17, 18]. In an attempt to reduce sample bias during the period of time immediately following the onset of the pandemic restrictions (T2^b^), only schools that participated at all three data collection waves were used (*N* = 78). Students with missing values for any variables of interest were removed for all analyses (8% of students in the cross-sectional samples had missing values and 9% of students in the longitudinal sample had missing values). All students included in the sample were under the legal age to purchase cannabis in their province. As shown in Fig. 1, the repeat cross-sectional analyses used student data from T1 (*n* = 38,890), T2 (T2^a^, *n* = 17,189; T2^b^, *n* = 6,920), and T3 (*n* = 22,795) and the longitudinal analyses used data from 4,677 students linked from T1 to T3. The T2^b^ sample was smaller than the T2^a^ sample given that the student-level participation rates within participating schools decreased from the typical 70–85% we have in COMPASS annually to 29% during T2^b^ data collection period during the immediate pandemic response period; this was largely driven by a transition to virtual learning (novel to schools and students at the time) which resulted in limited school coordination and student engagement within this particular wave only.

As described elsewhere [17, 18], the repeat cross-sectional analysis examined three waves of data from two cohorts of *schools*. Cohort-R1 included data from students attending schools where the T2 data were provided by paper-based surveys prior to the onset of the COVID-19 pandemic. Cohort-R2 was comprised of data from students attending schools where the T2 data were provided by online surveys immediately after the onset of the COVID-19 pandemic.

The longitudinal analysis examined data from two cohorts of *students*. Cohort-L1 included students from the longitudinal sample whose T2 data were provided by paper-based surveys prior to the onset of the COVID-19 pandemic. Cohort-L2 was comprised of students from the longitudinal sample whose T2 data were provided by online surveys immediately after the onset of the COVID-19 pandemic.

Evaluating the impact of the COVID-19 pandemic on youth behaviour and perceptions creates an interesting methodological challenge as data from a non-exposed control group are not available given the scope of the pandemic restrictions [22]. However, by taking advantage of the unique data available within COMPASS, we can still explore differences in perceptions of cannabis access between the cohorts (*schools* or *students*) where the T2 data collection bordered both pre- and post-pandemic onset periods. Examining differences between the two cohorts allows some insight to be gained as to the potential immediate impact of the COVID-19 pandemic on youth perceptions of cannabis access; the most robust design based on the data available.

### Measures

Given COMPASS is a prospective study that started in 2012, our measures of cannabis also use the term ‘marijuana’ to reflect common youth lexicon when referring to cannabis. Students were asked “Do you think it would be difficult or easy for you to get marijuana if you wanted some?”, with the following response options: difficult, easy, I do not know. Responses were recoded as follows [easy vs. (*ref*) difficult/I do not know]; since our primary concern focused on perceptions of access being easy, we purposefully collapsed I don’t know and difficult into one referent group since these respondents did not perceive access as easy. Respondents were also asked, “In the last 12 months, how often did you use marijuana or cannabis?” with the following response options: I have never used marijuana, I have used marijuana but not in the last 12 months, Less than once a month, Once a month, 2 or 3 times a month, Once a week, 2 or 3 times a week, 4 to 6 times a week, Every day. A student who reported they have never used cannabis was considered a never user, a student who reported using cannabis less than once a month or not in the last 12 months was classified as a non-current user, and a student who reported using cannabis one or more times a month was classified as a current user. Covariates included gender (female, male), grade (Secondary I/II, 9, 10, 11, 12), ethnicity (White, Black, Asian, Latin American/Hispanic, Other/Mixed), weekly spending money (none, $1-$20, $21-$40, $41-$100, more than $100, I don’t know), and province (British Columbia, Alberta, Ontario, Quebec).

### Analyses

Descriptive statistics were examined at each time point for the repeat cross-sectional sample, and for the baseline characteristics of each cohort in the longitudinal sample. Consistent with previous research [17, 18], for the repeat cross-sectional analysis, a logistic regression model using a generalized estimating equations (GEE) approach was used to obtain population-average odds of reporting ‘easy’ for perceptions of cannabis access while accounting for school-level clustering. The GEE approach is considered robust against the misspecification of the covariance structure [23]. A working exchangeable log odds ratio structure was also used. The odds of reporting that cannabis would be easy to access (Model 1) was examined by year, controlling for gender, grade, province, ethnicity, and spending money. An interaction effect between Cohort-R2 (Cohort-R1 as a reference group) and time (2018/19 as the reference group) was included in the models to examine potential distinct trends in perceptions of cannabis access being easy between the two cohorts of schools at each time point, keeping in mind that the data collections occurred at different times during T2 data collections between the two cohorts (immediately before the pandemic started and immediately after the pandemic started).

The longitudinal analysis was also consistent with previous research [17, 18], where we used a logistic regression model with a GEE approach to capture the population average odds of reporting that cannabis would be easy to access, while accounting for within-student association among the responses over time. A working exchangeable correlation structure was used. The odds of reporting that cannabis would be easy to access (Model 2) was examined by year and cohort, controlling for gender, baseline grade, province, ethnicity, and spending money. Interaction effects between Cohort-L2 (Cohort-L1 as a reference group) and time (2018/19 as the reference group) were also included in the model to examine potential distinct trends in reporting cannabis as easy to access between the two cohorts of students at each time point. All statistical analyses were performed using SAS 9.4.

## Results

As shown in Table 1, in the cross-sectional sample the prevalence of students reporting that cannabis was easy or difficult to access significantly changed over time (χ^2^ 1137.86, *p* < 0.001); students reporting that cannabis was easy to access decreased by 26.7% from 2018/19 (51.0%) to 2020/21 (37.4%). The prevalence of cannabis use also significantly changed over time (χ^2^ 594.45, *p* < 0.001); current cannabis use decreased by 40.9% from 2018/19 (12.7%) to 2020/21 (7.5%). In the longitudinal sample, the baseline prevalence of students reporting that cannabis was easy to access was slightly higher but not significantly different (χ^2^ 0.607, *p* = 0.4356) in Cohort-L1 (32.1%) relative to Cohort-L2 (31.0%). The prevalence of current cannabis use was also slightly higher but not significantly different (χ^2^ 2.561, *p* = 0.2779) in Cohort-L1 (3.3%) relative to Cohort-L2 (2.5%); note that the frequency of respondents reporting that accessing cannabis was easy and that they were current cannabis users were both lower in the longitudinal sample compared to the repeat cross-sectional samples.

**Table 1:** Descriptive statistics for the repeat cross-sectional and longitudinal samples used for examining trends in youth perceptions of cannabis access prior to and during the onset and progression of the COVID-19 pandemic, COMPASS study (2018/19, 2019/20, 2020/21)

	**Repeat Cross-sectional Sample**			**Longitudinal Sample**	
	**Time 1** **2018/19** *n*=38,890	**Time 2** **2019/20** *n*=24,109	**Time 3** **2020/21** *n*=22,795	**Cohort-L1**^c^ *n*=3,139	**Cohort-L2**^d^ *n*=1,538
	% (n)	% (n)	% (n)	% (n)	% (n)
Cohort					
Cohort-R1^a^	17,746 (45.6)	17,189 (71.3)	11,066 (48.6)	-	-
Cohort-R2^b^	21,144 (54.4)	6,920 (28.7)	11,729 (51.4)	-	-
Gender					
Female	19,258 (49.5)	12,701 (52.7)	12,262 (53.8)	1,819 (58.0)	1,027 (66.8)
Male	19,632 (50.5)	11,408 (47.3)	10,533 (46.2)	1,320 (42.0)	511 (33.2)
Grade				^Grade at Baseline^	^Grade at Baseline^
Secondary I/II^e^	8,269 (21.3)	5,580 (23.2)	6,513 (28.6)	1,171 (37.3)	815 (53.0)
9	9,067 (23.3)	5,626 (23.3)	5,138 (22.5)	1,289 (41.1)	525 (34.1)
10	8,733 (22.5)	5,285 (21.9)	4,891 (21.5)	679 (21.6)	198 (12.9)
11	8,185 (21.0)	4,876 (20.2)	4,084 (17.9)	-	-
12	4,636 (11.9)	2,742 (11.4)	2,169 (9.5)	-	-
Province					
Alberta	314 (0.8)	257 (1.1)	260 (1.1)	47 (1.5)	0 (0.0)
British Columbia	4,335 (11.2)	3,043 (12.6)	1,830 (8.0)	296 (9.4)	74 (4.8)
Ontario	16,709 (42.9)	9,338 (38.7)	8,149 (35.8)	1,143 (36.4)	353 (23.0)
Quebec	17,532 (45.1)	11,471 (47.6)	12,556 (55.1)	1,653 (52.7)	1,111 (72.2)
Ethnicity					
White	28,453 (73.1)	18,012 (74.7)	17,438 (76.5)	2,531 (80.6)	1,262 (82.1)
Black	1,422 (3.7)	600 (2.5)	595 (2.6)	39 (1.3)	42 (2.7)
Asian	3,302 (8.5)	2,082 (8.6)	1,592 (7.0)	205 (6.5)	78 (5.1)
Latin American/Hispanic	854 (2.2)	503 (2.1)	378 (1.7)	35 (1.1)	15 (1.0)
Other/Mixed	4,859 (12.5)	2,912 (12.1)	2,792 (12.2)	329 (10.5)	141 (9.1)
Weekly Spending Money					
None	6,066 (15.6)	4,107 (17.0)	5,176 (22.7)	560 (17.8)	284 (18.5)
$1 to $20	9,351 (24.1)	5,692 (23.6)	4,327 (19.0)	975 (31.1)	484 (31.5)
$21 to $40	4,363 (11.2)	2,474 (10.3)	1,767 (7.8)	352 (11.2)	176 (11.4)
$41 to $100	4,669 (12.0)	2,666 (11.1)	1,969 (8.6)	230 (7.3)	106 (6.9)
More than $100	7,474 (19.2)	4,379 (18.1)	4,506 (19.8)	251 (8.0)	106 (6.9)
I don’t know	6,967 (17.9)	4,791 (19.9)	5,050 (22.1)	771 (24.6)	382 (24.8)
Cannabis Use^f^					
Never use	28,879 (74.3)	18,985 (78.8) 2,735 (11.3)	18,710 (82.1)	2,892 (92.1)	1,432 (93.1)
Non-current use	5,052 (13.0)	2,389 (9.9)	2,374 (10.4)	143 (4.6)	68 (4.4)
Current use	4,959 (12.7)		1,711 (7.5)	104 (3.3)	38 (2.5)
Cannabis Access^g^					
Easy	19,848 (51.0)	10,406 (43.2)	8,521 (37.4)	1,009 (32.1)	477 (31.0)
Difficult or I don’t know	19,042 (49.0)	13,703 (56.8)	14,274 (62.6)	2,130 (67.9)	1,061 (69.0)

^a^Cohort-R1: students attending schools where the Time 2 data was collected using paper-based surveys prior to the onset of the COVID-19 pandemic

^b^Cohort-R2: students attending schools where the Time 2 data was collected using online surveys after the onset of the COVID-19 pandemic

^c^Cohort-L1: students in the longitudinal sample where their Time 2 data was collected using paper-based surveys prior to the onset of the COVID-19 pandemic

^d^Cohort-L2: students in the longitudinal sample where their Time 2 data was collected using online surveys after the onset of the COVID-19 pandemic

^e^Schools in Quebec only

^f^Students who reported “I have never used marijuana” are classified as ‘never users’, while those who reported “Once a month”, “2 or 3 times a month”, “Once a week”, “2 or 3 times a week”, “4 to 6 times a week”, and “Every day” are classified as ‘current users’. Those who reported “I have used marijuana but not in the last 12 months” or “Less than once a month”, are classified as ‘non-current users’

^g^Students were asked “Do you think it would be difficult or easy for you to get marijuana if you wanted some?”, with the following response options (easy, difficult or I do not know)

**Fig. 2:**
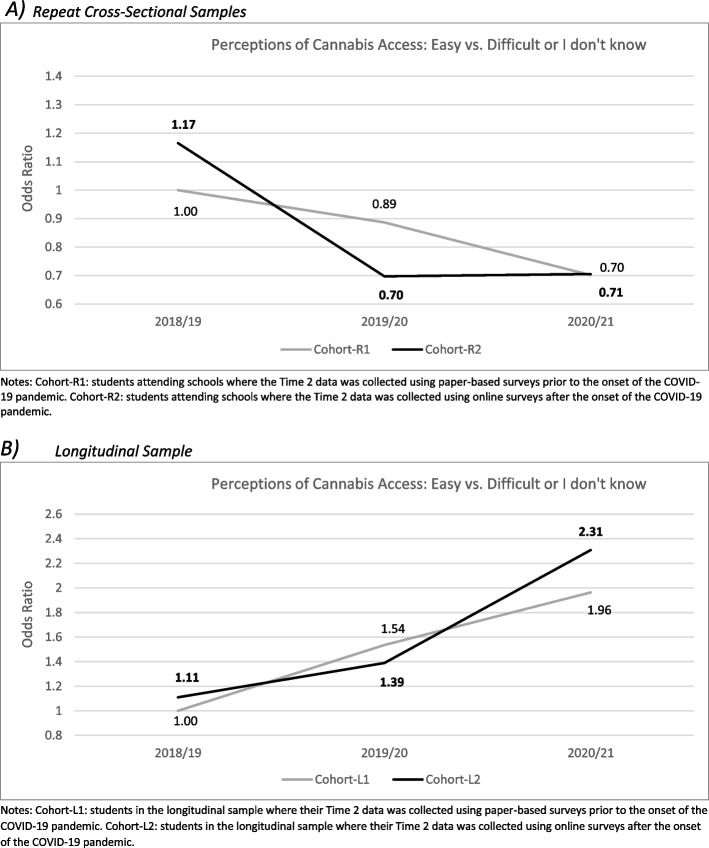
Generalized estimating equation parameter estimates for the interaction of Time and school Cohort when examining the likelihood of reporting that cannabis is easy to access within the repeat cross-sectional samples (**A**) and the longitudinal sample (**B**) from the COMPASS study (2018/19, 2019/20, 2020/21)

The GEE parameter estimates for the repeat cross-sectional samples (Model 1) and the longitudinal sample (Model 2) are both available in Table 2. As shown for Model 1, the likelihood of reporting that cannabis access was easy was substantially higher among non-current (β 1.60, *p* < 0.001) and current cannabis users (β 2.35, *p* < 0.001) relative to never users, and slightly higher among males (β 0.08, *p* < 0.001) relative to females, controlling for grade, weekly spending money, ethnicity and province. Although not shown in Table 2, relative to students in grade 9, students in grades 10 (β 0.39, *p* < 0.001), 11 (β 0.64, *p* < 0.001), and 12 (β 0.72, *p* < 0.001) were more likely to report that cannabis access was easy; younger students in Secondary I and II in Quebec (β -0.82, *p* < 0.001) were less likely to report that cannabis access was easy. A significant interaction between Cohort and Time was also identified. As shown in Fig. 2A, although perceptions of cannabis access being easy decreased across waves of the repeat cross-sectional sample, relative to students in Cohort-R1 in 2018/19, the likelihood of perceiving cannabis access as easy decreased significantly in Cohort-R2 in 2019/20 (immediately after the onset of the pandemic) and then remained stable in 2020/21 during the ongoing pandemic response, whereas the likelihood of perceptions of cannabis access being easy declined slightly in Cohort-R1 in 2019/20 prior to the onset of the pandemic and decreased further during the ongoing pandemic period in 2020/21. This suggests that in the repeat cross-sectional sample, perceptions of cannabis access being easy appears to have been significantly impeded during the initial pandemic period.

**Table 2:** Generalized estimating equation parameter estimates examining the likelihood of perceiving cannabis access as ‘easy’ prior to and during the onset and progression of the COVID-19 pandemic using the repeat cross-sectional samples (*n*=85,794) and the longitudinal sample (*n*=4,677) from the COMPASS study (2018/19, 2019/20, 2020/21)

	**Model 1**^a^**: Cannabis Access is Easy ** *Repeat Cross-sectional Sample*			**Model 2**^b^**: Cannabis Access is Easy** *Longitudinal Sample*		
	Estimate	95% CI	*p*-value	Estimate	95% CI	*p*-value
*Intercept*	-0.92	-1.04, -0.79	<0.001	-2.27	-2.58, -1.96	<0.001
Gender						
Male	**0.08**	0.02, 0.04	<0.001	-0.03	-0.13, 0.08	0.605
Female (ref)	-			-		
Grade^c^						
Secondary I or II^d^	**-0.82**	-0.91, -0.74	<0.001			
9	-					
10	**0.39**	0.35,0.44	<0.001			
11	**0.64**	0.58, 0.69	<0.001			
12	**0.72**	0.04, 0.65	<0.001			
Cannabis Use^e^						
Never use (ref)	**-**			-		
Non-current use	**1.60**	1.52, 1.68	<0.001	**1.07**	0.94, 1.20	<0.001
Current use	**2.35**	2.24, 2.46	<0.001	**1.88**	1.70, 2.07	<0.001
Time						
2018/19 (ref)	-			-		
2019/20	**-0.12**	-0.16, -0.08	<0.001	**0.43**	0.35, 0.51	<0.001
2020/21	**-0.35**	-0.43, -0.28	<0.001	**0.67**	0.59, 0.76	<0.001
Cohort^f^						
Cohort-1 (ref)	-			-		
Cohort-2	**0.15**	0.04, 0.27	<0.01	0.10	-0.03, 0.24	0.137
Time/Cohort Interaction						
2018/19 * Cohort-1 (ref)	-			-		
2018/19 * Cohort-2	-			-		
2019/20 * Cohort-1	-			-		
2019/20 * Cohort-2	**-0.39**	-0.48, -0.31	<0.001	**-0.20**	-0.35, -0.06	<0.01
2020/21 * Cohort-1	-			-		
2020/21 * Cohort-2	**-0.15**	-0.24, -0.05	<0.01	0.06	-0.10, 0.21	0.434

^a^Model 1: Cannabis access is easy (*n*=38,775) vs. cannabis access is difficult or I don’t know (*n*=47,019), controlling for grade, weekly spending money, province and ethnicity

^b^Model 2: Cannabis access is easy (*n*=1,486) vs. cannabis access is difficult or I don’t know (*n*=3,191), controlling for grade at Time 1, weekly spending money at Time 1, province and ethnicity

^c^In the longitudinal model this is grade at baseline (2018/19)

^d^Quebec sample only

^e^Students who reported “I have never used marijuana” are classified as ‘never users’, while those who reported “Once a month”, “2 or 3 times a month”, “Once a week”, “2 or 3 times a week”, “4 to 6 times a week”, and “Every day” are classified as ‘current users’. Those who reported “I have used marijuana but not in the last 12 months” or “Less than once a month”, are classified as ‘non-current users’

^f^Cohort-1 represents students attending schools where the 2019/20 data was collected using paper-based surveys prior to the onset of the COVID-19 pandemic (September to March 10, 2020) and Cohort-2 represents students attending schools where the 2019/20 data was collected using online surveys after the onset of the COVID-19 pandemic (April to July, 2020)

As shown for Model 2, the likelihood of reporting that cannabis access was easy was higher among current cannabis users (β 1.88, *p* < 0.001) and slightly higher among non-current cannabis users (β 1.07, *p* < 0.001) relative to never users, controlling for grade at baseline, weekly spending money, ethnicity and province; gender was not significantly associated with perceptions of cannabis access in the longitudinal model. A significant interaction between Cohort and Time was also identified. As shown in Fig. 2B, although perceptions of cannabis access being easy significantly increased over time in both Cohort-L1 and Cohort-L2, respondents in Cohort-L2 had a smaller increase in likelihood of perceiving cannabis access as easy relative to students in Cohort-L1 at T2 but then experienced a larger increase in the likelihood of perceiving cannabis access as easy relative to students in Cohort-L1 at T3. This suggests that in the longitudinal sample, perceptions of cannabis access being easy appears to have been impeded during the initial pandemic period but then there was a rebound effect during the ongoing pandemic period.

## Discussion

While there has been a growing number of studies focused on examining changes in cannabis use among Canadian youth since the onset of the Cannabis Act [2, 24–26], and more recently since the onset of the COVID-19 pandemic [7, 9, 18], there appears to be a paucity of research dedicated to examining changes in youth perceptions of cannabis availability over the same period of time. In response, this study provides unique and novel evidence of how youth perceptions of cannabis access have changed since the onset of the Cannabis Act and throughout the early and ongoing COVID-19 pandemic response period using both repeat cross-sectional and longitudinal data. This is important as it has been suggested that the Cannabis Act is failing to adequately restrict youth access to cannabis given the ongoing high prevalence of underage Canadian youth who report using cannabis [27]; data from the 2019 National Cannabis Survey also show that accessing cannabis from illegal sources remains common in Canada post-legalization [25]. Our data suggest that in our large samples of youth, perceptions of cannabis access as being easy has declined in prevalence since legalization and through the early and ongoing pandemic response periods, and that the likelihood of perceiving cannabis access as easy increases with age and having experience using cannabis.

More research examining the issue of youth perceptions of cannabis availability is required as not only is cannabis perceived as more socially acceptable following liberalizing policy changes (e.g., legalization), but youth who report their ability to access cannabis as being easy are also at greater risk for using cannabis [13]. Although it has been suggested that youth under the legal age to purchase cannabis do not appear to experience barriers to obtaining cannabis within legalized contexts [6], our data suggest that perceptions of availability are changing. Our cross-sectional results highlighted that since the onset of legalization and throughout the early and ongoing pandemic periods, the prevalence of underage youth reporting that cannabis would be easy to access has been declining. While this may be an indicator that components of the Cannabis Act are effectively limiting the availability of cannabis to underage youth, we are unable to discern if it may have also been impacted by the onset of the COVID-19 pandemic restrictions that limited peer network socialization opportunities given that peers represent one of the most common sources by which underage youth access cannabis [4, 5]. Regardless, at least the declines observed align with evidence from youth in the US showing that perceptions of cannabis being easy to access has been declining (2002–2015) [13]. It also appears to tangentially align with representative data from seven quarters of the National Cannabis Survey (NCS) showing declines in illegally sourced cannabis among Canadians from the pre- to post-legalization periods (51.7% to 40.1% respectively) [25]. Regardless of the promising decline observed, considering that more than a third of youth in the 2020/21 sample still reported it would be easy to access cannabis if they wanted some, ongoing efforts to limit underage youth access to cannabis are still required. It would be beneficial for future research to identify the contexts surrounding youth that endorse such perceptions, or the individual characteristics associated with such perceptions (or ultimately who they access cannabis from and how), to inform the tailoring and targeting of future interventions associated with cannabis control efforts aligned with the successful ongoing implementation and enforcement of the Cannabis Act.

Considering the risk of cannabis use often increases with age [28–30], few youth cannabis users quit during their high school years [26, 31], and evidence from emerging adults and adults highlighted that use of cannabis typically increased among users during the early stages of the pandemic [11, 12], our longitudinal finding that likelihood of reporting it would be easy to access cannabis as students progressed through high school was not surprising. It is important to note that this finding occurred through both waves of data collected during the early and ongoing pandemic response periods, suggesting increasing age was more relevant than the pandemic impact with respect to perceptions surrounding the ability to access cannabis among youth in our sample. Despite the Cannabis Act clearly stating that no person may sell or provide cannabis to any person under the age of 18 and there are two criminal offenses related to providing cannabis to youth with maximum penalties of 14 years in jail [1], prior to the pandemic youth commonly report not being concerned about being arrested or having fear of being caught with cannabis as enforcement does not appear to be a priority for police [32]. The Cannabis Act currently allows underage youth to possess up to the equivalent of 5 g of dried cannabis [1], and while this provision may help to ensure underage youth are not arrested for possessing small amounts of cannabis, it has been suggested that it creates an opportunity for underage youth to possess cannabis which can then also be shared with peers [33]. Data from the 2017 Canadian Cannabis Survey (CCS) suggest that among youth cannabis users, the most common sources of cannabis pre-legalization were sharing with or buying from friends and peers [5]. Considering there was no significant difference in perceptions of access between males and females in our longitudinal model, future cannabis control efforts may want to prioritize the development and implementation of youth-focused interventions targeted to different age groups and tailored to those with or without prior experience using cannabis.

### Limitations

This study is subject to some important limitations. For instance, data are not available in the COMPASS study to examine why youth believe cannabis is easy or difficult to access, or to examine the source(s) underage youth use to access cannabis or believe that they could easily access cannabis if they wanted some. While the COMPASS study is based on self-reported data, which can be prone to recall and social desirability biases, it uses passive consent protocols that limit self-selection and response bias, particularly for substance use measures [34], where youth may have concerns about reporting participation in potentially illicit behaviour; student names are not required for longitudinal data linkage, helping to preserve perceptions of anonymity for honest reporting which is important for the topic of investigation. COMPASS is also based on a convenience sample of participating schools, so results may not be generalizable to all Canadian youth. Similar to limitations reported in previous research using COMPASS to examine the impact of the COVID-19 pandemic on behavioural outcomes [17, 18], due to the nature of the data available it is not possible to have comparison group data for this natural experiment as there is no non-exposed control group during this global pandemic; the interrupted time series non-experimental design we used that incorporated both repeat cross-sectional and longitudinal data with relatively large sample size spanning the pre-pandemic to early and ongoing pandemic periods would be considered our best available study design [22]. Another possible COVID-19 related limitation includes the transitioning from school-based paper-and-pencil questionnaires to online assessment, which may have influenced reports but was unavoidable given the constraints imposed by COVID-19 restrictions on data collection protocols and was adjusted for in our models by the Cohort indicator variable.

## Conclusion

Given the lack of existing evidence, our robust examination of changes in perception of cannabis access during the early and ongoing pandemic period provides valuable new insight to the literature. We identified that while the prevalence of youth reporting that cannabis is easy to access has declined since legalization and throughout the early and ongoing pandemic periods, the likelihood of underage youth reporting that cannabis is easy to access increases as students age and progress through high school. Given that more than a third of respondents in the most recent wave of COMPASS data reported that cannabis was easy to access, there is substantial room for continued cannabis control efforts to have an impact.

## Data Availability

The datasets used and/or analysed during the current study available from the corresponding author on reasonable request submitted via the following online application form (https://uwaterloo.ca/compass-system/sites/ca.compass-system/files/uploads/files/compass_data_use_application_2020.pdf).

## Abbreviations

COMPASS: The Cannabis use, Obesity, Mental health, Physical activity, Alcohol use, Smoking, and Sedentary behaviour Study
GEE: Generalized Estimating Equation
